# Feasibility of At-Home Hand Arm Bimanual Intensive Training in Virtual Reality: Case Study

**DOI:** 10.2196/57588

**Published:** 2024-09-06

**Authors:** James E Gehringer, Anne Woodruff Jameson, Hailey Boyer, Jennifer Konieczny, Ryan Thomas, James Pierce III, Andrea B Cunha, Sandra Willett

**Affiliations:** 1 Virtual Reality Laboratory Munroe-Meyer Insitute University of Nebraska Medical Center Omaha, NE United States; 2 Department of Physical Therapy Munroe-Meyer Insitute University of Nebraska Medical Center Omaha, NE United States; 3 Department of Occupational Therapy Munroe-Meyer Insitute University of Nebraska Medical Center Omaha, NE United States; 4 Physical Therapy Program Department of Kinesiology Colorado Mesa University Grand Junction, CO United States

**Keywords:** cerebral palsy, HABIT, home intervention, virtual reality, rehabilitation, VR, case study, hand, hands, arm, arms, intensive training, feasibility, game, games, gaming, hand arm bimanual intensive training, motor, movement, home setting, home-based, child, children, male, males, men, quasi-experimental, parent, parents, intervention, interventions

## Abstract

This single-participant case study examines the feasibility of using custom virtual reality (VR) gaming software in the home environment for low-dose Hand Arm Bimanual Intensive Training (HABIT). A 10-year-old with right unilateral cerebral palsy participated in this trial. Fine and gross motor skills as well as personal goals for motor outcomes were assessed before and after the intervention using the Box and Blocks Test, Nine-Hole Peg Test, and Canadian Occupational Performance Measure. Movement intensities collected via the VR hardware accelerometers, VR game scores, and task accuracy were recorded via the HABIT-VR software as indices of motor performance. The child and family were instructed to use the HABIT-VR games twice daily for 30 minutes over a 14-day period and asked to record when they used the system. The child used the system and completed the 14-hour, low-dose HABIT-VR intervention across 22 days. There was no change in Box and Blocks Test and Nine-Hole Peg Test scores before and after the intervention. Canadian Occupational Performance Measure scores increased but did not reach the clinically relevant threshold, due to high scores at baseline. Changes in motor task intensities during the use of VR and mastery of the VR bimanual tasks suggested improved motor efficiency. This case study provides preliminary evidence that HABIT-VR is useful for promoting adherence to HABIT activities and for the maintenance of upper extremity motor skills in the home setting.

## Introduction

Unilateral cerebral palsy (CP) is a neurological condition that is present from birth or acquired within the first year of a child’s life [1]. It is characterized by abnormalities of tone, muscle weakness, sensory or perceptual deficits, and motor incoordination of the affected side, which can lead to significant impairments of motor function result. Physical and occupational therapy focuses on motor learning tasks that incorporate the intensive, repetitive, and progressively challenging practice of goal-oriented functional tasks that improve upper extremity motor function for children with unilateral CP [2,3].

Hand Arm Bimanual Intensive Training (HABIT) is a therapeutic approach for unilateral CP that focuses on coordinated, bimanual, and functional movements of the upper extremities [4-14]. As a therapy with a robust foundation of evidence, HABIT uses principles of neuroplasticity and motor learning or control to enhance hand and arm function in children with unilateral CP [15]. A high-intensity block of HABIT therapy, or 30-90 hours within a 2-week period of time, is the documented “dose” necessary to enhance bimanual ability, unilateral dexterity, self-care function, and achievement of functional motor goals post intervention [4-6,11-13,16-20]. Despite its effectiveness, implementation of HABIT is primarily confined to short-term, intensive camp-like settings [20]. The structure of these intensive camps presents challenges in the ongoing implementation of HABIT due to financial constraints, therapists’ availability, scheduling time with children or families, and transportation barriers [8-10]. Further, the positive effects of HABIT, particularly in improving the functional use of the affected hand and arm, persist only 3 months postintervention, then decline by 6 months post intervention [21]. Given the decline in functional abilities post-HABIT intensives, innovative home-based strategies are essential to help children with unilateral CP access interventions like HABIT and maintain functional motor gains.

Virtual reality (VR) is a therapeutic tool poised to transform interventions like HABIT. Offering an immersive, engaging environment that replicates real-life scenarios for functional movement, VR provides an interactive and motivating “gaming” environment that potentially enhances adherence and access to therapeutic interventions. Multiple studies demonstrate the effectiveness of VR games in improving gross motor function, balance, gait, and reaching kinematics in children with CP [22-27]. Our interdisciplinary team developed HABIT-VR, a therapeutic VR software that incorporates motor learning principles and gamifies bimanual upper extremity HABIT tasks into portable environments that are scalable to individual ability levels [12].

This innovation has several advantages over traditional HABIT camp: (1) it gamifies therapy thus potentially enhancing client motivation and interest; (2) it addresses potential frustration or fatigue through automated task scaling, for example, making the task harder or easier based on the participant’s ongoing successes or failures; and (3) it is readily available for use in the home environment. The ultimate goal of this home-based HABIT-VR is to create an optimal motor challenge. Modifications to task-specific parameters, such as accuracy or speed, precisely scale the challenge point, and, in theory, foster both increased motor learning and task engagement [28-31]. While early investigations suggest HABIT-VR’s success when integrated with traditional HABIT treatment, further exploration is crucial to determine its feasibility as a remote therapy tool [12].

Past attempts at therapy gamification have used commercialized video games [32-36]. Such games do not have the customization needed to scale the game to the child’s unique motor ability. Accessibility options change difficulty levels globally, when it may be more appropriate to scale a specific challenging aspect. This leads to games that are either too difficult or too simple to promote the child’s interest [37]. Motor learning principles essential to the success of HABIT, such as scaling of task difficulty, intrinsic and extrinsic motivators, and variable motor practice, are important for positive therapeutic outcomes. Most gamified therapies to date have not integrated these features [37]. Further, for a study examining the feasibility of use at home, these motor learning features need to be automatically scaled based on performance, to allow for the maintenance of an optimal challenge point, sustained participant use, and dynamic task constraint variations [37]. HABIT-VR aims to address these issues through patient performance tracking, all without requiring in-person intervention.

The purpose of this case study is to present the outcomes of a 10-year-old child with right unilateral CP who participated in low-dose, one-game HABIT-VR in their home following discharge from a conventional physical therapy plan of care. We hypothesized that the use of HABIT-VR in the home environment would lead to improvements in upper-extremity motor performance and an individual’s perceived occupational performance. Secondarily, this investigation explores ways to analyze upper extremity activity using actigraphy data (ie, a wearable device used to measure physical activity levels and rest cycles over time) to better understand changes in upper extremity motor control while using VR.

## Methods

### Ethical Considerations

The Institutional Review Board at the University of Nebraska Medical Center reviewed and approved the protocol (0491-20-EP) for this investigation. The procedures followed were in accordance with the ethical standards of the responsible committee on human experimentation and with the Helsinki Declaration of 1975, as revised in 2000. The participant and his guardian provided informed assent and consent, respectively.

### Participant

The child (“M”) in this study was a 10-year-old male with unilateral CP, left hand dominant, who was classified as Gross Motor Function Classification System level 1, Manual Ability Classification System level 1. “M” had just been discharged from an episode of outpatient PT services. “M” had prior video game experience and tried HABIT-VR games periodically to provide developers with end user perceptions and suggestions for games.

### HABIT-VR Development for Home Use

**Figure 1:**
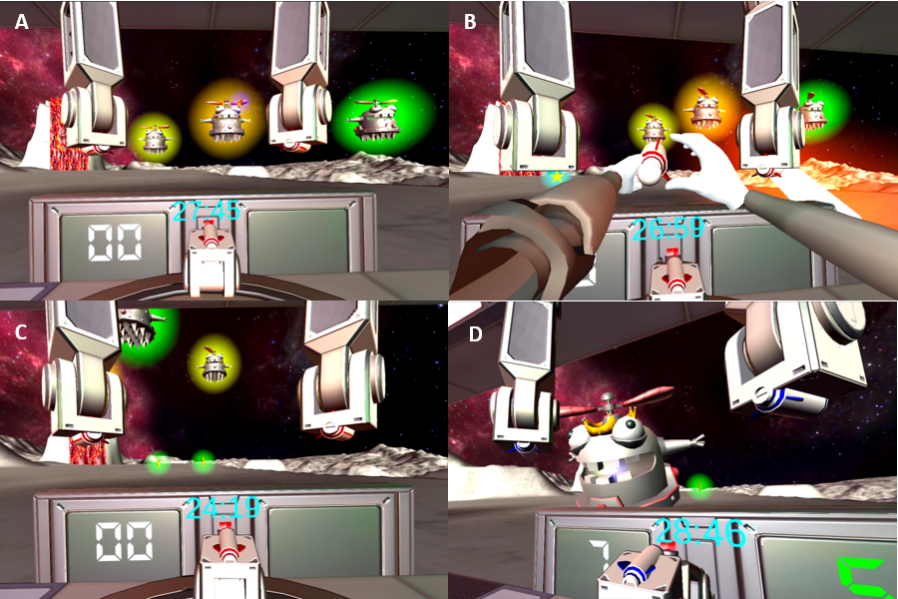
Hand Arm Bimanual Intensive Training virtual reality (HABIT-VR) single game. (A) The participants played a single HABIT-VR game that tasked them with using rockets to fire at robots. (B) Rockets were activated by touching the back of the rocket while holding the rocket with the other hand. Rockets would only fire when one hand was holding the rocket and the other was touching the back. (C) Power-ups would show up occasionally, in this image the green orbs with stars in them, which were designed to encourage the user. (D) Once the user beat their high score, the boss battle would begin.

This investigation strategically automated various game features to align with critical motor learning principles and enhance the home-based use of a single HABIT-VR game (Figure 1).

The HABIT approach provides intensive, child-focused repetitive practice by offering a structured practice embedded in play and functional activities requiring the use of both hands while maintaining a child-friendly approach [6]. It uses key principles of motor learning and neuroplasticity such as practice specificity, types of practice, feedback, repetition, increasing movement complexity, and motivation [11]. These key principles were integrated into the HABIT-VR game. The game gradually increases in complexity, requiring children to use both hands repetitively and in progressively challenging ways. By incorporating tasks such as retrieving rockets and hitting targets, we ensure that the activities are engaging and need bimanual use. The game was also designed to create a fun and immersive experience that would keep children motivated and engaged while practicing repetitive bimanual activities

Specifically, in this single HABIT-VR game, the player is tasked with shooting robots with rockets (Figure 1A). The player holds the Meta Quest controller to move the VR hands in 3D space. The position and rotation of the controller are tracked via infrared cameras embedded in the Meta Quest VR headset. The player must pick up a rocket, by moving one hand and controller over the rocket’s location and pressing a controller trigger. Once the trigger is pressed and held, an activation point appears at the back of the rocket (Figure 1B). Using the hand and controller pair that is not holding the rocket, the player can move over the location of the activation point to launch the rocket. The rocket then flies linearly in the forward direction. If the rocket makes contact with a robot after it is fired, a point is added to the score. The long-term task in this game is to beat a target score, which is the player’s high score plus 5%. If the target score is beaten, then the boss battle is activated, which is a single larger robot that needs to be hit multiple times in quick succession (Figure 1D). All these activities are to be performed within 30 minutes, and if 30 minutes pass, the game exits automatically.

Task demands were graded to allow for success, and difficulty was progressed with the child’s performance improved by requiring greater speed or accuracy and based on our previous experience [12]. By incorporating automated difficulty scaling, motivators, and variable practice elements, the investigation aimed to maintain optimal challenge points, sustained participant use, and diversified task constraints without necessitating in-person intervention [37]. This approach not only ensures a more engaging and effective home-based intervention but also holds promise for broader applications in motor learning contexts. Finally, remote data monitoring of hand and arm function through 3D accelerometers in the VR hardware was added to explore the use of actigraphy as a measure of movement characteristics. Actigraphy uses accelerometer-based devices to monitor and quantify physical activity levels and movement patterns over time. By wearing an actigraph device, individuals can track their daily activity levels, including intensity of movement, duration of physical activity, and periods of rest or sedentary behavior. The device records movement data continuously providing a detailed overview of activity patterns throughout the day and night. Overall, actigraphy in the context of movement analysis offers a noninvasive and objective method for monitoring physical activity and movement patterns, which can be valuable for various research, clinical, and rehabilitation purposes.

The automation of difficulty scaling was achieved through real-time participant performance tracking. Each attempt at the designated task, launching a rocket, was recorded by the software. Successful rocket launches, resulting in hits on target robots, were categorized as successes, whereas instances where rockets were fired but targets were not hit were considered failures. The software then analyzed the success rate based on the last set of attempts within a specific time frame. If the participant’s success rate exceeded a predefined threshold (60%), a global increase in difficulty level was initiated. Consequently, the speed of the target was increased, and its size was reduced, thereby enhancing the demands on visual-motor tracking, timing, and motor skills. Conversely, if the success rate fell below another predefined threshold (30%), the global difficulty level was decreased, resulting in slower targets with larger sizes. The game encompassed 5 distinct difficulty levels, with each session commencing at level 1. The adjustments in target speed and size between difficulty levels were governed by predefined percentages (25% for speed and 10% for size). It is noteworthy that these difficulty levels were designed to ensure that targets remained within the realm of achievability, thus mitigating the potential for participant discouragement arising from unachievable challenges [38-41].

Simple motivators were incorporated into the game in the form of a high-score tracker, a target score mechanism, and rocket power-ups. The high-score tracker and target score served a dual purpose: providing participants with specific performance objectives and triggering in-game events. The game perpetually tracked the participant’s high score and set the target score at a level 5% higher than the current high score. This design strategy consistently encouraged participants to strive for improved performance. To further incentivize participants to attain the target score, the culmination of the game, symbolized by the “boss battle,” was unlocked exclusively upon reaching the target score. The ensuing boss battle will be discussed in greater detail later. Additionally, rocket power-ups were introduced to the game, offering 4 unique enhancements: bigger rockets, target freeze, rapid-fire, and triple rockets. These power-ups were integrated to align with motor learning and neuroplasticity principles by supporting the value of novel or surprise-driven learning experiences [42,43]. Power-ups were randomized within the game environment, requiring the participant to hit them with rockets to activate their benefits. In addition to their unexpected nature, these power-ups were designed to encourage curiosity and exploration, thereby serving as additional motivational factors during the early phases of the game.

Variable practice was instilled within the game through the use of the “boss battle” scenario and the associated power-ups. While the standard targets necessitated a single rocket hit and moved along all 3 dimensions, the “boss battle” featured a solitary, sizable target that exclusively moved horizontally within the participant’s field of view. However, this boss target demanded a rapid succession of 7 rocket hits for successful completion. Maintaining momentum was crucial; a rocket hitting the boss within a 3-second interval of the preceding hit preserved progress, but intervals exceeding 3 seconds necessitated a restart. This design shift emphasized the speed of rocket firing, diverting focus from target accuracy and precision. Additionally, the power-ups, denoted by stars, introduced variable practice as they were activated by hitting smaller stationary targets. Although hitting these stationary targets required higher accuracy, the absence of movement obviated the extensive reliance on visuo-motor tracking skills.

HABIT-VR may also hold significant potential for providing meaningful and quantifiable feedback on changes in the user’s hand or arm motor function, both to the user and remotely to the clinician. Recent endeavors have used actigraphy to objectively quantify motor changes [44-46] and have been used to measure changes in upper extremity use in children with unilateral CP [16]. Importantly, these measures could be integrated within HABIT-VR, as the data necessary for their calculation can be collected through the sensors in the VR headset. The integration of these measures presents a valuable opportunity for objective evaluation of changes in hand and arm function, as these measures could be reported back to treatment teams remotely to quantify changes, enhancing both the clinical relevance and applicability of HABIT-VR in therapeutic home settings.

### Motor Function Assessment

A case study design was used. The participant attended 3 baseline (Phase A) assessment sessions, each spaced a week apart, starting 3 weeks before the onset of intervention. The posttraining assessment took place 1 week after the 14-hour treatment threshold was reached. Pre- and posttraining assessments included the box and blocks test (BBT), and the Nine-hole peg test (NHPT). These clinical assessments were chosen to mirror assessments commonly used to evaluate the effects of HABIT [3,8,16,30,31]. The Canadian Occupational Performance Measure (COPM) is a semistructured interview designed to set goals and capture a client’s self-perception of occupational performance in the areas of self-care, productivity, and leisure. It has been used to measure self-efficacy in children with CP, which is an individual’s belief that they can perform a task and that they have the capacity to change how they perform to succeed [47]. This has been used to measure self-efficacy in the motor domain in children with CP and in VR environments VR training is thought to improve self-efficacy in children with CP as it may provide a greater sense of accomplishment [47].

The BBT was administered as standard, with 2 trials per arm. The BBT is a tool to measure unilateral gross manual dexterity in children with CP. It has a documented, intraclass correlation coefficient (ICC) of 0.98, a minimal detectable change of 5.95, and a minimal clinically important difference ranging from 5.29 to 6.46 [48]. The NHPT was performed with a 50-second time limit, as defined by the Shirley Ryan Ability Lab [49]. The NHPT is considered a valid and reliable tool to measure fine manual dexterity in children with CP, with high intrarater reliability (ICC of 0.94 for the nonaffected side and ICC of 0.96 for the affected side), a minimal detectable change of 4 s for the dominant side and 12 s for the nondominant side [50].

### Task Performance Measures

Motor learning measures captured through the VR software included the following: the number of targets hit, which is the game score, and task accuracy. The software added 1 point to the game score every time a target was hit. This score was recorded and visible to the participant in the game as a motivator for the participant, to assess skill performance and progression, and for goal setting to maintain a correct challenge point. The participant was tasked with matching a target score every session, which was set to 105% of their high score. In addition, each rocket launched was tracked, so that task accuracy could be calculated.

### Actigraphy Measures

The 3D movement accelerometry data were collected at 120 Hz using the accelerometers in the Meta Quest controllers used to control the game. These data were collected during the preassessments, the at-home intervention phase, and the postassessments. Activity counts through actigraphy were selected to explore the use of accelerometry measures. These data were then down-sampled to 30 Hz and reformatted into the correct CSV format to be processed via Actilife (version 6.13.5; ActiGraph Corporation) and transformed into movement intensities (activity level per session: light physical activity [LPA] vs moderate to vigorous physical activity [MVPA]). In Actilife, the standard activity count processing pipeline used Evenson youth cutoffs. While Evenson youth cutoffs were established based on neurotypical adults, these cutoffs have been validated for children with CP [51,52].

### Intervention

During the intervention phase (Phase B), “M” used the VR device and played one of the HABIT-VR games at home over a 3 weeks’ time frame. The participant and their parents were instructed to play the single HABIT-VR game twice a day, 30 minutes per session, for a total of 1 hour per day. They were asked to do this for 14 consecutive days, for a total of 14 hours of treatment. This is considered the minimum amount of time needed to see motor learning changes [53]. Adherence to recommended dosage was tracked in two ways, (1) with a family log of dates or times used and (2) with the VR software.

### Data Analysis

Analyses were conducted using R (R Foundation for Statistical Computing), using the Single-Case Data Analyses for Single and Multiple Baseline Designs (scan) package [54]. Data were analyzed according to 3 phases: preintervention, during the intervention, and postintervention. Cohen *d* was used to quantify the effect size of changes within game scores, firing rates, accuracy rates, and movement intensities. Cohen suggested that *d* of 0.2 be considered a “small” effect size, 0.5 represents a “medium” effect size, and 0.8 a “large” effect size [55]. The standard deviation considers variations within individual participants and can be used to derive an estimate across cases for a standardized change in level [56].

## Results

### Adherence to Recommended Intervention Parameters

**Figure 2:**
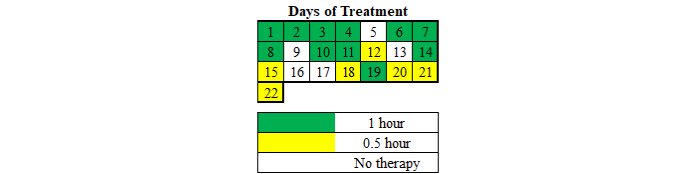
Calendar representation of Hand Arm Bimanual Intensive Training virtual reality (HABIT-VR) use. Each number represents the number of days since the participant was given HABIT-VR to use at home. Green days represent days that the participant used HABIT-VR for 1 hour per day. Yellow days represent days that HABIT-VR was used for half an hour per day. White days represent days that HABIT-VR was not used.

Total dose was 14 hours across 22 days, with 11 days adhering to the intervention recommendations, 5 days with a half dosage, and 6 days skipped (Figure 2). These data were reported by both the HABIT-VR software and the log the participant maintained.

### Clinical Measures

COPM goals were established and focused on goals regarding walking skills and school participation that include upper-extremity skills. Goal 1 was regarding pain when running, goal 2 focused on participation on their basketball team, goal 3 focused on participation in a physical education class, goal 4 focused on walking at home without an assistive device, and goal 5 focused on stair climbing speed. Preintervention, the average COPM performance and satisfaction scores were 7.8 (SD 1.2) and 7.4 (SD 1.4), respectively (Table 1). Postintervention, the average COPM performance and satisfaction scores were 9.4 (SD 0.8) and 9 (SD 1.3) respectively.

**Table 1:** Canadian Occupational Performance Measure scores. Each row is an individual skill the participant wanted to work on. Scores in the first columns are preintervention, while scores in the second columns are postintervention scores.

	Performance		Perception	
	Preintervention	Postintervention	Preintervention	Postintervention
Goal 1	8	10	7	10
Goal 2	6	8	5	7
Goal 3	7	9	8	8
Goal 4	9	10	9	10
Goal 5	9	10	8	10

### Box and Blocks

For the dominant hand, the average box and blocks score during preassessment was 60.5 (SD 2.6) blocks; postassessment, 65 blocks (Table 2). For the nondominant hand, the preassessment average was 50.8 (SD 2.1) blocks; postassessment, 51.5 blocks (Table 2).

**Table 2:** Box and Blocks scores pre- and postintervention scores. The preintervention data represent the average of the 3 preintervention scores. The postintervention score was the 1 postsession score. Both tests were performed with the dominant and nondominant hand. The measurement is the number of blocks moved.

	Preintervention, mean (SD)	Postintervention
Dominant	60.5 (2.6)	65
Nondominant	50.8 (2.1)	51.5

### Nine Hole Peg Test

For the dominant hand, the average 9-hole peg test score for the preassessment was 0.91 (SD 0.03) pegs/second; postassessment, 0.92 pegs/second (Table 3). For the nondominant hand, the preassessment average was 0.57 (SD 0.05) pegs/second; postassessment, 0.62 pegs/second (Table 3).

**Table 3:** Nine Hole Peg test scores before and after the intervention. The preintervention data represent the average of the 3 preintervention scores. The postintervention score was the 1 postsession score. Both tests were performed with the dominant and nondominant hand. The measurement is the number of pegs placed per second.

	Preintervention, mean (SD)	Postintervention
Dominant	0.91 (0.03)	0.92
Nondominant	0.57 (0.05)	0.62

### Task Performance

**Figure 3:**
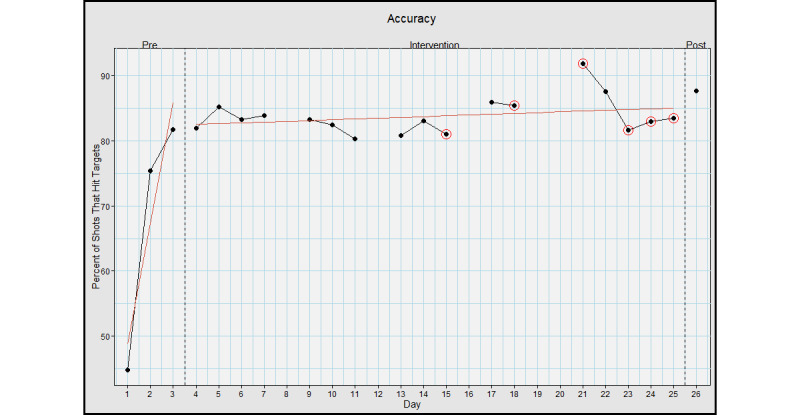
Task accuracy when using Hand Arm Bimanual Intensive Training virtual reality (HABIT-VR). This graph represents the percentage of rockets fired when using HABIT-VR that hit a target. The y-axis represents the percentage of rockets fired that hit a target. The x-axis represents each day the participant used HABIT-VR including the pre- and postintervention assessments. Days circled in red represent the days where the participant only used HABIT-VR for half an hour. Blank days are the days the participant did not use HABIT-VR. The red line represents the trend of the values for the respective phase.

The average preintervention task success rate was 67.3% (SD 19.8%), the intervention average was 83.8% (SD 2.8%), and the postassessment success rate was 87.6%, with an effect size (*d*) of 1.17; large effect (Figure 3).

**Figure 4:**
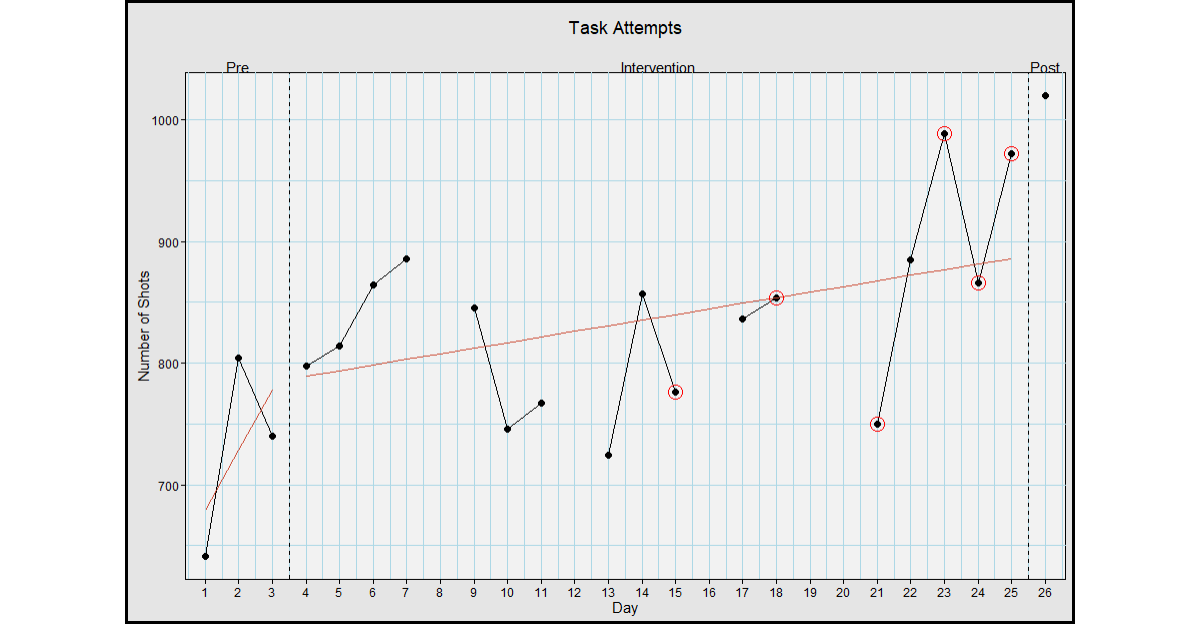
Task attempts when using Hand Arm Bimanual Intensive Training virtual reality (HABIT-VR). This graph represents the number of rockets fired when using HABIT-VR. The y-axis represents the absolute number of rockets fired. The x-axis represents each day the participant used HABIT-VR including the pre- and postintervention assessments. Days circled in red represent the days where the participant only used HABIT-VR for half an hour. Blank days are the days the participant did not use HABIT-VR. The red line represents the trend of the values for the respective phase.

The average preintervention number of attempts was 728.3 (SD 82.1) shots, the intervention average was 837.0 (SD 74.0) shots, and the postassessment number of attempts was 1020.0 shots, with an effect size (*d*) 1.39; large effect (Figure 4).

**Figure 5:**
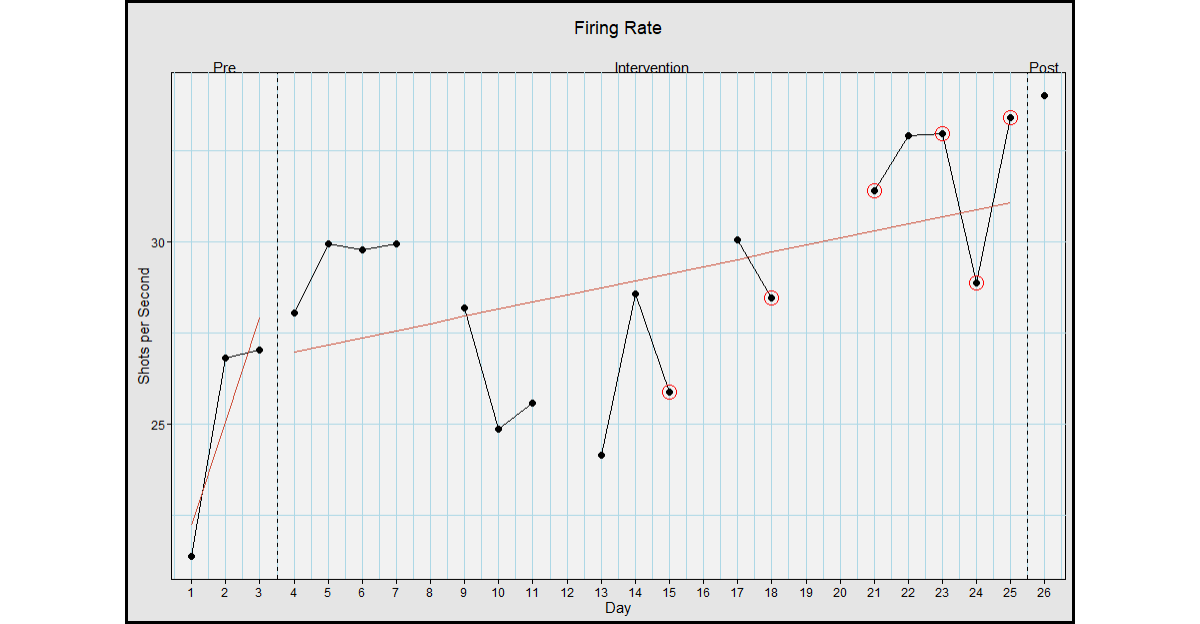
Firing rate when using Hand Arm Bimanual Intensive Training virtual reality (HABIT-VR). This graph represents the rockets fired per minute when using HABIT-VR. The y-axis represents the rockets per minute fired. The x-axis represents each day the participant used HABIT-VR including the pre- and postintervention assessments. Days circled in red represent the days where the participant only used HABIT-VR for half an hour. Blank days are the days the participant did not use HABIT-VR. The red line represents the trend of the values for the respective phase.

The average preintervention firing rate was 25.1 (SD 3.2) shots per minute, the intervention average was 29.0 (SD 2.8) shots per minute, and the postassessment firing rate was 34.0 shots per minute, with an effect size of *d* 1.31; large effect (Figure 5).

### Actigraphy

**Figure 6:**
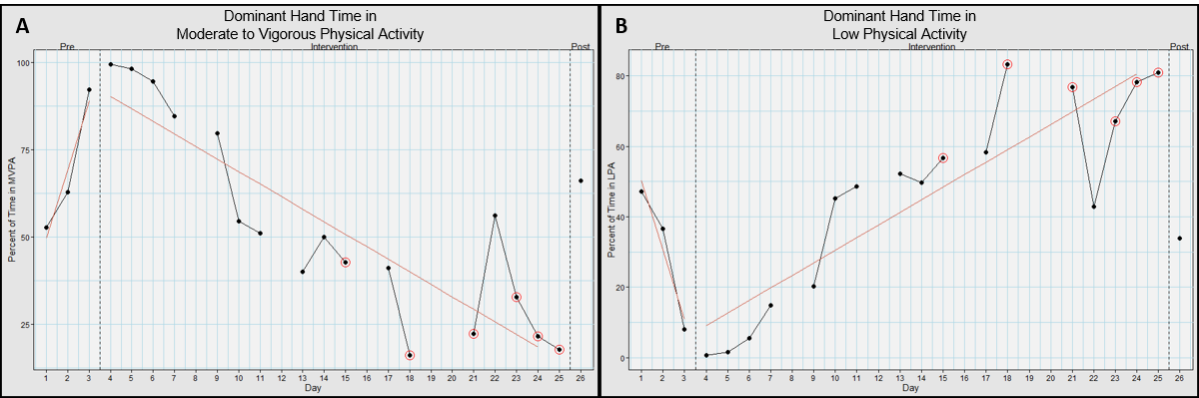
Dominant hand movement intensities when using Hand Arm Bimanual Intensive Training virtual reality (HABIT-VR). This graph represents the percentage of time the dominant hand spent in (A) moderate to vigorous physical activity and (B) low physical activity when using HABIT-VR. The y-axis represents the percentage of time in the respective activity level. The x-axis represents each day the participant used HABIT-VR including the pre- and postintervention assessments. Days circled in red represent the days where the participant only used HABIT-VR for half an hour. Blank days are the days the participant did not use HABIT-VR. The red line represents the trend of the values for the respective phase. MVPA: moderate to vigorous physical activity.

For the dominant hand, it spent 69.2% (SD 16.7%) time in MVPA in baseline, 53.1% (SD 27.7%) time in MVPA in intervention, and 66.1% time in post (Figure 6A). This had an effect size (*d*) of 0.649; medium effect.

For the dominant hand, it spent 30.6% (SD 16.6%) time in light physical activity at baseline, 46.1% (SD 27.4%) time in light physical activity in intervention, and 33.9% time in post (Figure 6B). This had an effect size (*d*) 0.629; medium effect.

**Figure 7:**
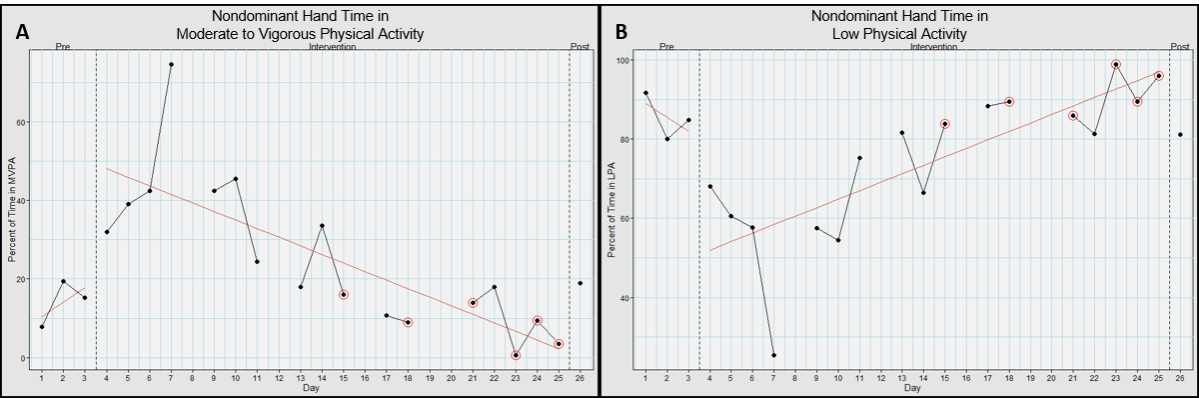
Nondominant hand movement intensities when using Hand Arm Bimanual Intensive Training virtual reality (HABIT-VR). This graph represents the percentage of time the nondominant hand spent in (A) moderate to vigorous physical activity and (B) low physical activity when using HABIT-VR. The y-axis represents the percentage of time in the respective activity level. The x-axis represents each day the participant used HABIT-VR including the pre- and postintervention assessments. Days circled in red represent the days where the participant only used HABIT-VR for half an hour. Blank days are the days the participant did not use HABIT-VR. The red line represents the trend of the values for the respective phase.

For the nondominant hand, it spent 14.2% (SD 4.8%) time in MVPA in baseline, 25.5% (SD 18.5%) time in MVPA in intervention, and 18.8% time in post (Figure 7A). This had an effect size (*d*) 0.801; large effect.

For the nondominant hand, it spent 85.5% (SD 4.8%) time in light physical activity at baseline, 74.2% (SD 18.3%) time in light physical activity in intervention, and 81% time in post (Figure 7B). This had an effect size (*d*) 0.812; large effect.

## Discussion

This study examined the feasibility of using a single, customized HABIT-VR game in the home. The main outcomes were adherence to the protocol, changes in upper-extremity gross and fine motor clinical measures, self-perception of occupational performance, and VR task performance. Additionally, this investigation explored the use of actigraphy data recorded from the VR hardware to quantify and understand changes in motor activity of the upper extremity. These results suggest that HABIT-VR may be a therapeutic option in the home environment that may maintain upper extremity motor function over time and between episodes of care. Collectively, results underscore the potential impact of HABIT-VR at home on both clinical measures and objective kinematic assessments.

Adherence to home programs is one way of understanding the usefulness of a therapeutic program [57]. Adherence to the recommended VR gaming protocol exhibited variations over this study’s duration, extending beyond the initially planned 14 days to conclude after 22 days. The participant attributed the absence of therapy in the early phase to reported illness and personal events, while a period of waning interest, due to playing the same game repeatedly, during the middle phase contributed to the days with a single 30-minute session. However, the participant emphasized that the incorporation of a high-score function motivated interaction with the game. Notably, the participant suggested that the inclusion of additional games or environments within the single game could have enhanced their experience. Future at-home studies should explore the use of the complete suite of HABIT-VR games to determine long-term effects on both adherence and motor outcomes.

The maintenance of clinically relevant upper extremity motor outcome measures following a low-dosage home treatment in our study is noteworthy. This finding holds particular significance considering the potential for children with unilateral CP to experience a decline in functional abilities post discharge. The ability of low-dosage, at-home HABIT-VR gaming to sustain performance over a 1-month period is noteworthy. This study provides preliminary evidence suggesting that HABIT-VR may serve as a valuable intervention tool between in-clinic sessions, potentially bridging the therapeutic gap. Recognizing that 60% of children aged 8 to 18 years engage in approximately 2.5 hours of video game activity daily [58], the integration of low-dose HABIT-VR into leisure time is a feasible strategy to help maintain the functional gains achieved through in-clinic intensives.

The COPM ratings increased in our participant, a positive outcome considering the prevalent issue of self-efficacy in children with CP. Self-efficacy, an individual’s assessment of their ability to learn and perform skills in specific situations, has been a focal point in previous studies using therapeutic video game systems with children with CP [47]. These studies report improvements in COPM scores, emphasizing the significance of a VR play environment in providing children with CP with enjoyable, age-appropriate activities. Our participant reported enhanced performance and satisfaction. While the average COPM score did not exhibit a clinically relevant 2-point change, this may be attributed to a ceiling effect with initial scores being high. The average of both performance and perception scores improved by 1.6 points, with 3 skills showing at least a 2-point increase in perceived performance and satisfaction. Also, of note for the COPM: the 2-point minimal clinically important difference was originally established based on data from adults in a mental health facility [59] and recent works suggest the relevant change may be different for different populations and contexts [60-62]. Indeed, the change observed in this trial may highlight that technological interventions like HABIT-VR can positively impact self-perception among individuals with CP.

Skill performance, assessed through task success rates, task attempts, and firing rate, demonstrated an improvement. Specifically, the task success rate improved from an average preintervention success rate of 67.3% (SD 19.8%) to 87.6% at postassessment. Task attempts and firing rate follow similar trends, with a 40% and 35.5% increase from the pre- to postintervention phase, respectively. These data reflect typical motor learning patterns, with a sharp increase early on, and incremental increases in later learning [63-66]. The sharp increase is typically indicative of the fast motor learning stage [65,67], while the incremental increases seen during intervention represent slow motor learning [64,68,69]. This suggests that motor learning occurs while using HABIT-VR. Overall, the participant increased their performance of the task by over 30% for all performance metrics, demonstrating a high level of mastery of the single HABIT-VR game.

Actigraphy measures revealed high levels of movement intensity initially, followed by a decrease in the second half of the study. This reduction in movement intensity may indicate increased boredom with the single-game HABIT-VR. The participant reported decreased interest in the game as the study progressed, and in turn, movement intensities may have decreased. However, when combined with the number of attempts and firing rate while playing the game, it seems that the participant was playing the game at a higher performance level with reduced physical exertion. This suggests improved movement efficiency with a practiced motor task. With either interpretation, actigraphy measurements serve as a useful measure for automated detection of therapeutic gaming use and motor function. Integrating open-source options directly into therapeutic software provides clinicians with objective, measurable data for decision-making [70]. Further, integrating these measures into the VR hardware device eliminates the need for an additional device to record these data, reducing research overhead, reducing participant burden, and allowing for remote monitoring of home programs.

Despite insights gained from this study, certain limitations are acknowledged. The use of a case study design, particularly within the context of the Manual Ability Classification System level I, restricts the generalizability of our findings to a broader population. While the detailed exploration of a single case provides in-depth insights, caution must be exercised in extrapolating these results to individuals with varying levels of manual ability. Additionally, it is crucial to note that the analysts were not blinded, potentially introducing bias into the assessment process. Future studies using larger sample sizes and incorporating blinded analyses would enhance the validity and reliability of the findings. Another limitation is the singular postintervention data collection. There is potential for variation between data collection sessions, as demonstrated in the preintervention data, so a singular data collection postintervention may not capture the full change in outcome measures. Future studies should include additional postintervention data collections to ensure a more robust postintervention measure. Further, this investigation did not include a direct measure of bimanual function. While we were able to measure the function of both hands independently, future studies should include measures of bimanual function, such as the Assisting Hand Assessment. Finally, this investigation only used a single HABIT-VR game. This single-game setup has the participant focusing on a single set of movements, and not the whole array of movements that HABIT-VR or traditional HABIT would have a child working on. It may have affected the participant’s level of interest. Future investigations should explore how these outcomes change when additional HABIT-VR games are included.

This investigation provides preliminary evidence supporting the feasibility of HABIT-VR treatment as an at-home therapeutic option. These results suggest that HABIT-VR may be an innovative approach to bridge the gap between intensive in-clinic sessions, such as traditional HABIT, or between active treatment phases. Moreover, the utility of actigraphy as a measure for both performance and use emerges as a valuable contribution, providing clinicians with objective data about adherence and motor changes that could be monitored remotely.

## Abbreviations

BBT: Box and Blocks Test
COPM: Canadian Occupational Performance Measure
CP: cerebral palsy
HABIT: Hand Arm Bimanual Intensive Training
MVPA: moderate to vigorous physical activity
NHPT: Nine-Hole Peg Test
VR: virtual reality
